# Influence of Cementation on the Aesthetical Appearance of Full-Ceramic Restorations

**DOI:** 10.3390/ma16031236

**Published:** 2023-01-31

**Authors:** Jörg Meister, Natalia Kaschuba, Michael Romer, Christoph Bourauel

**Affiliations:** 1 Department of Periodontology, Operative and Preventive Dentistry, Bonn University, 53111 Bonn, Germany; 2 Center of Applied Medical Laser Research and Biomedical Optics (AMLaReBO), Bonn University, 53111 Bonn, Germany; 3 Laser Application in Medical Sciences Research Center, Shahid Beheshti University of Medical Sciences, Tehran 19839 69411, Iran; 4 Oral Technology, Bonn University, 53111 Bonn, Germany; 5 Independent Researcher, 53111 Bonn, Germany

**Keywords:** optical analysis, visible and NIR laser, light transmission, light scattering, full-width at half maximum (FWHM), sample thickness

## Abstract

The use of dental ceramics as restorative materials requires corresponding luting materials (cements) that, in turn, influence the visual appearance of the restoration. Due to the high light transmission through the ceramics, the cements can affect the color perception of the dental restoration. This study aims to investigate the optical effects of various cements on the visual appearance of full-ceramic restorations. Three fixing polymer resins (Bifix SE (VOCO GmbH, Cuxhafen, Germany), Breeze^TM^ (Pentron Clinical, West Collins Orange, CA, USA), and Panavia^TM^ F. 2.0 (Kuraray, Noritake, Osaka, Japan)), with layer thicknesses of 50, 100, 200, and 250 µm, were applied onto a ceramic base model (0.4 mm thick), and irradiated with laser light of wavelengths 532, 632.8, and 1064 nm. Light intensities and scattering effects of light of various wavelengths were angle-dependent, analyzed using a goniophotometer with perpendicular light incidence on the sample specimen (base model plus luting material). In addition, the transmitted power of the light through the sample specimen was determined as a function of the layer thickness. With increasing layer thickness, power losses of respectively 30% for Bifix SE and Breeze^TM^ in the visible spectral range were comparable, whereas Panavia^TM^ F. 2.0 showed a power loss of ca. 44% here. For the near-infrared range, the power losses for all cements were 25%. This could be confirmed by the interpretation of the line widths. Moreover, the line widths for thin cement layer thicknesses (50 and 100 µm) in the visible spectral range displayed only a redistribution of light by scattering, which does not affect color perception at all. In addition, at 200 and 250 µm, absorption occurred which causes a change in color perception. Within the scope of this study, it could be shown that for thin-layer thicknesses of the cement applied here, there is no adverse optical effect on the aesthetic visual appearance of the restoration.

## 1. Introduction

The past years have seen a considerably rising prevalence in metal-free dental restorations, keeping with the trend towards personalized aesthetics. Consequently, the visual appearance in using dental ceramics as full-fledged restorative materials requires establishing corresponding fixing techniques that, on the one hand, compensate for the mechanical stresses and, on the other hand, support optical characteristics. The aesthetics can only then be predicted when the optical interactions of all involved components of the restoration (here, ceramic including the luting cement plus tooth enamel) are considered.

The tooth represents a complex optical system. Its natural appearance is determined by color, translucency, opacity, as well as opalescence (sheen) and fluorescence, and is marked by the combination of all these characteristics. The incident light on the tooth enamel is partially reflected on the surface and subsequently is refracted, partially absorbed, and scattered. Due to the structural elements—the enamel prisms which conduct light as well as refract it (dispersion)—the enamel layer displays a high degree of light transmission until the enamel–dentin junction (interface) is reached. In dentin, the transmitted fraction of light is further refracted, selectively absorbed by the organic components, and is scattered, which ultimately causes how the tooth color is perceived extrinsically.

The special optical feature of dentin here is scattering, whereby the scattering of light on the dentin tubules exhibits light-conducting characteristics [1,2,3,4,5]. The whole optical impression of the tooth is ultimately affected by the surface morphology, thickness of the enamel layer, degree of mineralization, and discoloring of various types. Thus, in restorative measures, regarding aesthetics, the determination of the tooth color is essentially important, besides the restoration of masticatory function, bite position, and phonetics [6,7].

In modern dentistry, dental ceramics have proved themselves reliable with respect to morphology, surface texture, color reproduction, and durability [8,9]. What is especially advantageous for such restorations is the good biocompatibility, acid resistance in the mouth environment, low plaque depositions, suitable mechanical strength, as well as the possibility of restoring the natural optical/aesthetic properties [10,11,12]. Finally, the high lifespan also contributes towards preferentially using full-ceramic restorations clinically [13,14].

The bonding of the ceramic prothesis with the carrier material (tooth structure) is crucial for the lifespan. The purpose of the dental prosthesis, the material properties of the ceramic, as well as the clinical baseline situation altogether determine the type of the fixation. This includes, for example, the use in the front or lateral tooth area, mechanical stress parameters of the ceramic, as well as the morphological characteristics of the prepared tooth such as the stump height, depth of the preparation boundary, dimension of the preparation angle, and static and dynamic occlusion concepts [15]. Thus, upon selecting the fixing technique, one needs to consider ensuring the stability with respect to mastication and the sealing of the joining between the tooth and the restorative prosthesis, along with aesthetic aspects as well.

Full-ceramic materials, such as lithium disilicate ceramics, glass-infiltrated, and conventional oxide ceramics, which show mechanical strength values of over 350 MPa, can be fixed with a glass ionomer cement or zinc oxide phosphate cement. Another way to definitely fix the full-ceramic dental prosthesis is using luting composites. Having mechanical strength values of below 350 MPa (feldspar ceramics), the adhesive fixation is used for minimally invasive restorations such as veneers or Maryland bridges [16,17].

With respect to the chemical composition, luting composites resemble conventional polymers. The polymer matrix, consisting of methacrylate-based resins, is mixed with acrylic acid esters and complemented with initiators and stabilizers. Monomers with a small molecular mass are added for increasing the flow rate. Thus, the thin fluid consistency enables a precise incorporation of the dental restoration. Here, adhesive or self-adhesive or self-etching luting composites are employed. For the adhesive luting composites, a conditioning of the tooth structure is necessary. Then, the adhesive fixation takes place via the micromechanical anchoring and the chemical reaction between the luting material and the tooth structure [15,18]. To improve adhesion and depending on the material, the surfaces of the restorative ceramics typically are etched with hydrofluoric acid or are conditioned with aluminum oxide and silicon oxide particles via sandblasting [19].

### Optical Properties of Restorative Materials

Aligning the restorative materials onto the natural structures is an important aspect regarding aesthetic appearance. Nowadays, the translucency as well as the opacity of ceramic materials can be adapted to the clinical baseline situation. An increase in the crystallization in the matrix causes, for example, an increase in the opacity, thereby allowing a masking of discolored tooth stumps. Variations of the refractive index between the glass and crystalline phases, as well as the crystalline structure, grain size, pigmentation, number, size, and distribution of intrinsic defects, as well as the porosity of the ceramics all influence the light scattering and refraction, which ultimately affect the translucency and opacity and, thus, the light transmission [20]. Moreover, the surface preparation influences the optical properties of the ceramic prosthesis. Heffernan et al. [21] reported about an increase in the translucency of full-ceramic materials after the glazing. Thus, highly translucent silicate ceramics have established themselves in the production of veneers and crowns in the front-tooth area [22]. Consequently, together with the ceramics, the fixing materials need to create a neutral background to ensure the replication of the natural overall appearance.

Using laser light of wavelengths in the visible (VIS) and near-infrared (NIR) spectral ranges, this current work aims to investigate the effects of layer thicknesses of various luting polymers (cements) on the optical properties of full-ceramic restorations. The assumption that the layer thickness of the luting polymers used has no optical influence on the aesthetical appearance of the restorations is defined as a null hypothesis.

## 2. Materials and Methods

### 2.1. Sample Selection and Preparation

Within the scope of this study, three different luting polymers (cements) were chosen:

- Bifix SE (VOCO GmbH, Cuxhafen, Germany)—a self-adhesive, polymer-based, dual-cure luting cement—consists of bifunctional methacrylates, acidic methacrylates, and inorganic fillers with a filler material fraction of 70% by weight. The selected color shade was “Universal” [23].
- Breeze^TM^ (Pentron Clinical, West Collins Orange, CA, USA) is likewise a self-adhesive, polymer-based, dual-cure luting cement. This cement was used in the color shade “A2” [24].
- Panavia^TM^ F 2.0 (Kuraray, Noritake, Osaka, Japan) is a self-adhesive, polymer-based, dual-cure luting cement in a two-component mixing. The first component contains Methacryloyloxydecyl Dihydrogen Phosphate (MDP)-monomers and a mixture of dimethylacrylates as well as fillers, catalysts, and initiators. The second component likewise consists of dimethylacrylates as well as barium glass and various additives. The total amount of inorganic filling material is approx. 59% by volume. The particle size of the filler ranges from 0.04 µm up to 19 µm. The color shade “Light” was chosen for this study [25].

For determining the optical properties, the cements were applied to a restoration (base) model of a fine-structured feldspathic ceramic. The feldspathic ceramic “Cerec Blocs” (Dentsply Sirona, Charlotte, NC, USA) is characterized, among others, by a high translucency [26], which distinguishes it as a base model for this study. According to the Vita-reference, the color 2M2C with the color saturation Medium (M) and the middle brightness (Level 3) was used [26]. With a precision diamond belt saw (Exakt Advanced Technologies GmbH, Norderstedt, Germany), 45 ceramic discs with a thickness of 0.40 mm ± 0.05 mm could be won as base models from the “Cerec Bloc” (size: 12·14·18 mm). Before the cements were applied, the base models were pretreated on one side with 5% hydrofluoric acid, marketed as Vita Ceramics Etch (Vita Zahnfabrik, Bad Säckingen, Germany). Altogether, the combinations of the various cements with the base models subsequently formed the sample specimens for these studies.

**Figure 1:**
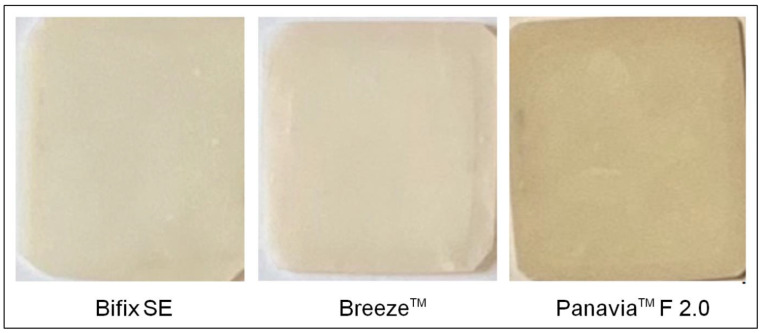
Sample specimen with a cement layer thickness of 200 µm, presented according to its perceived brightness of “light” (Bifix SE), over “medium” (Breeze^TM^), to “dark” (Panavia^TM^ F 2.0).

For producing the sample specimens with cement thickness layers of 50, 100, 200, and 250 µm, the gap size between two glass plates was measured using a feeler gauge. The cements were mixed according to manufacturer specifications, applied onto the base models, and fixated between both glass plates. By using a polymerization lamp (420–480 nm) of type Acton Mini LED^TM^ Standard (Acton Germany GmbH, Düsseldorf, Germany), the cements were cured at an intensity of 2000 mW/cm^2^ and at a total illumination time of 60 s. Here, the illumination took place in two steps, i.e., for 30 s through the glass plate and for 30 s after the glass plate was removed. The final thickness of the sample specimens was examined with an external micrometer of type Mitutoyo IP65 (Mitutoyo Deutschland GmbH, Neuss, Germany). Figure 1 exemplarily shows the sample specimens with a cement layer thickness of 200 µm, presented according to their perceived brightness.

Ultimately, 12 samples (three examined materials at four layer-thicknesses) were available for the laser optical studies. A ceramic base model was used as the reference.

### 2.2. Laser Sources

Lasers emitting light of various wavelengths were used for covering a selective color spectrum. The following lasers were used: a frequency-doubled Nd:YVO_4_ laser of type MGL-III (Changchun New Industries Optoelectronics Tech. Co. LTD., Changchun, China) emitting light of wavelength 532 nm (green) with a maximum power output of 250 mW; a helium-neon laser (He-Ne) emitting light of wavelength 632.8 nm (red) at 5 mW maximum power output (Research Electro-Optics Inc., Boulder, CO, USA); and a Nd:YVO_4_ laser emiting light of wavelength 1064 nm (near-infrared, NIR) at 1 W maximum power output (Changchun New Industries Optoelectronics Tech. Co. LTD., Changchun, China).

### 2.3. Experimental (Goniophotometer) Setup

Starting from the position of the sample specimen, all optical components such as the laser beam source, beam splitter, imaging and deflection optics, adjusting aperture, and sample and reference detector were aligned in one plane (optical beam path). Beam splitter, adjusting aperture, imaging and deflection optics, as well as the reference detector were positioned fixedly on the optical bench. Depending on the applied source of radiation, the laser beam is coupled in the optical beam path via folding mirrors. The adjusting aperture serves to align the laser beam onto the sample specimen and the sample detector. By means of the beam splitter and the imaging optics, the laser radiation is aligned to the reference detector, whereby intensity fluctuations that occur during the measurement can be compensated for. The sample specimen was clamped into the sample holder with a limited amount of spatial mobility. This mobility ensured that the irradiation of the sample specimen on various areas took place respectively under perpendicular incidence of light. With the help of a stepping motor (C-633 Mercury Step, Physik Instrumente, Karsruhe, Deutschland) and the Software LabVIEW 8.6 (LabVIEW, National Instruments, Austin, TX, USA), the sample detector can be aligned, computer-controlled, at an increment of 0.1° fully around the sample specimen (360°), thus recording the transmitted and reflected light. Photodiodes of type J16-5SP (Teledyne Judson Technologies, Montgomeryville, PA, USA) were used as the reference and sample detectors. Here, this deals with germanium photodiodes which can be used in the wavelength range of ≤600 nm and 1600 nm. At a distance of 13.5 cm between the sample specimen and sample detector and a detector area of 6.6 mm^2^, the light intensity distribution can be spatially detected under an aperture angle of 3°.

### 2.4. Irradiation Procedure

Power measurements and evaluation:

All power measurements were performed with an energy/power meter LabMax Top (Coherent, Santa Clara, CA, USA) combined with a PM10 detector (Coherent, Santa Clara, CA, USA). Moreover, an instrument-internal statistical data or trend analysis allowed the determination of mean and standard deviation values over a timed measurement period.

To ensure the comparability of all measurements, the laser powers of the vanadate lasers were adapted to the output power of the He-Ne laser (5 mW). Within the measuring period of 10 s, the average power was 4.70 mW ± 0.26 mW for all laser systems. Analogously, the measurement was performed on the ceramic base model (thickness 0.4 mm) as well as on every sample specimen with 50, 100, 200, and 250 µm cement thickness including the recording of all mean and standard deviation values. Hereby, the samples were positioned at best closely to the detector area so as to minimize possible scattering losses. Moreover, all sample specimens were irradiated from the ceramic side.

### 2.5. Line Width Measurements and Evaluation

**Figure 2:**
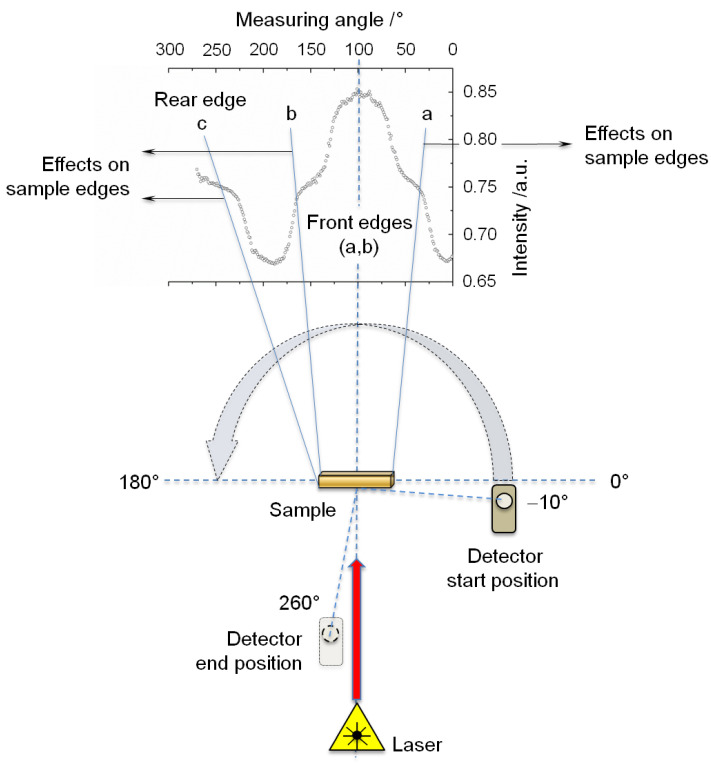
Illustration of the measurement principle for determining the line shapes and line widths Full Width at Half Maximum (FWHM). The diagram shows the intensity distribution of the transmitted light exemplified by the laser irradiation at 532 nm on a ceramic base model as a function of the measuring angle. The lines a, b, and c delineate edge effects which appear upon the interaction of the laser light with the base model.

With regard to line shape and line width (Full Width at Half Maximum (FWHM)), conclusions can be drawn regarding the optical properties of the material to be examined. As for the power measurements, all sample specimens were irradiated from the ceramic side. For determining the reflected and transmitted fractions of radiation on the sample specimens as well as on the base model, the sample detector was guided counter-clockwise around the test specimen in 1°-increments in the angular range of −10° (Start position) up to 260° (End position) (Figure 2).

To exclude manufacturing-related inhomogeneities in the cements, every sample specimen and base model underwent, respectively, four measurements at various spatial positions. By taking into account the number of samples and base model as well as the three laser wavelengths, this resulted in a total of 156 measurements lasting 80 min each. All measurements were conducted in a darkened room so as to rule out external light effects.

All measurement values were recorded as text files and transferred to Microsoft MS Excel 2010 (Microsoft Ireland Operations Ltd., Dublin, Ireland). Mean and standard deviation values were determined for every measurement. OriginPro 8.6 (OriginLab Corporation, Northampton, MA, USA) was used for creating graphs as well as determining the line widths Full Width at Half Maximum (FWHM).

### 2.6. Statistical Evaluation

Descriptive statistics, including calculation of the mean values, standard deviations, and an error estimation, were applied to the power measurements as well as for determining the line width measurements. For data evaluation, Microsoft MS Excel 2010 (Microsoft Ireland Operations Ltd., Dublin, Ireland) was used. The results were evaluated graphically using the spreadsheet calculation program OriginPro 8.6 (OriginLab Corporation, Northampton, MA, USA). Polynomial, exponential, and linear fit functions were used for evaluating and visualizing the data behaviors.

An explorative statistical analysis was inappropriate for this basic research.

## 3. Results

### 3.1. Power Measurement/Calibration and Measurement Results

**Figure 3:**
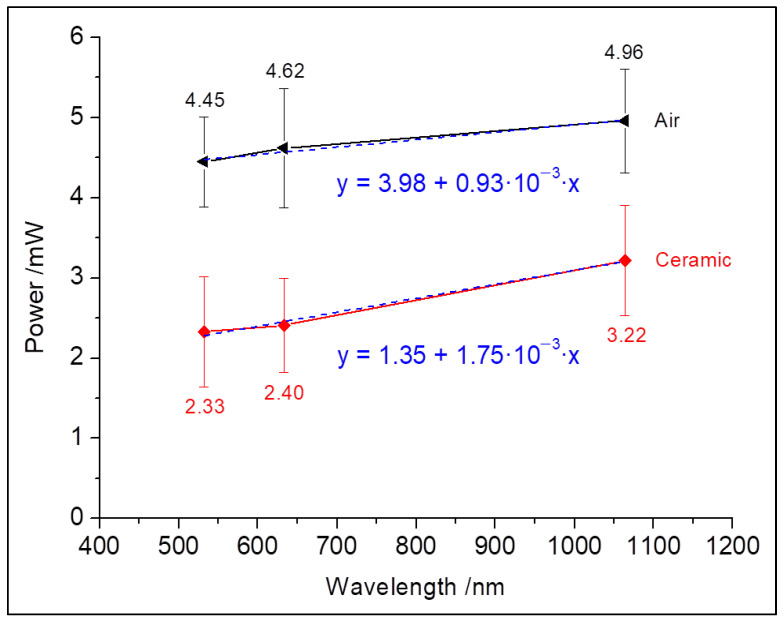
Adaptation of the laser output powers to the He-Ne laser (632.8 nm) in air (upper graph) as well as at the transmitted powers of all laser through the ceramic base model (lower graph). Also shown are the corresponding gradients of both graphs.

The measurements on the base model enabled an estimation of the influence of the carrier ceramic on the transmission behavior of the entire sample specimen. Figure 3 shows the measured laser output power in air as well as on the base model. Here, the mean transmitted power is 2.65 mW ± 0.50 mW. The ceramic should allow one to expect a uniform attenuation of the laser radiation (parallel shifting to lower laser output powers) over the entire wavelength range. However, the power reductions between the visible (ΔP = 2.1 W_532 nm_, ΔP = 2.2 W_632_._8 nm_) and near-infrared radiation (ΔP = 1.7 W_1064 nm_) showed clear differences which are also noticeable in the slopes of the linear fit functions.

**Figure 4:**
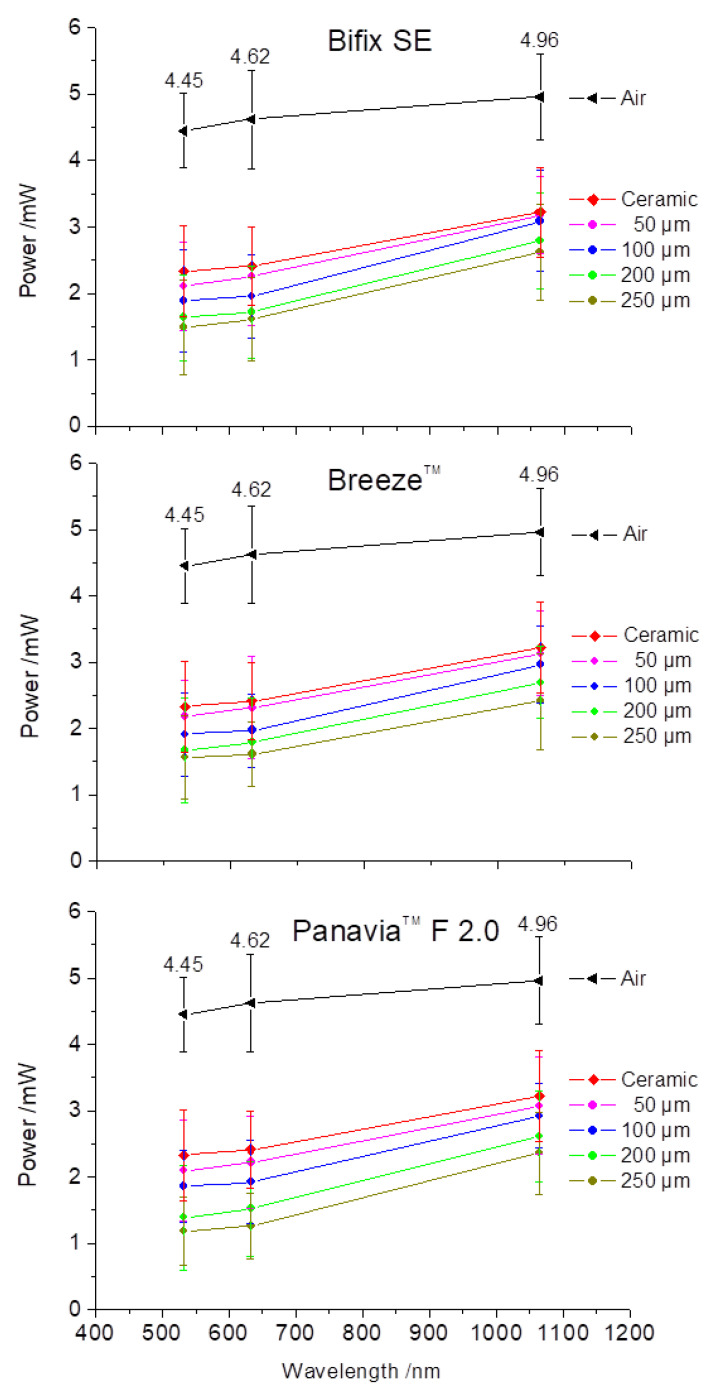
Influence of the layer thickness of the examined cements on the transmitted powers for the wavelengths 532, 632.8, and 1064 nm. For direct comparison, the adapted laser powers in air (black curve) as well as the transmitted powers of all the lasers through the ceramic base model (red curve) are shown.

Figure 4 shows the influence of the cement layer thickness on the transmitted laser powers for the corresponding laser wavelengths. Since the luting cements were applied onto the base model, the power values for all cement materials lie below the transmission curve of the base model. Moreover, with increasing layer thickness, there is a decrease in the transmitted power for all cements. Over the examined spectral range, an almost parallel power reduction could be observed for all examined materials upon increasing layer thickness. However, for the mean difference values Δ mW, there was a 10-times greater rise in the power from the visible to the near-infrared range for all cements. This illustrates a tendency towards increased transmission behavior of the incident radiation in the near-infrared range (Table 1).

### 3.2. Line Width Measurements/Calibration and Measurement Results

**Figure 5:**
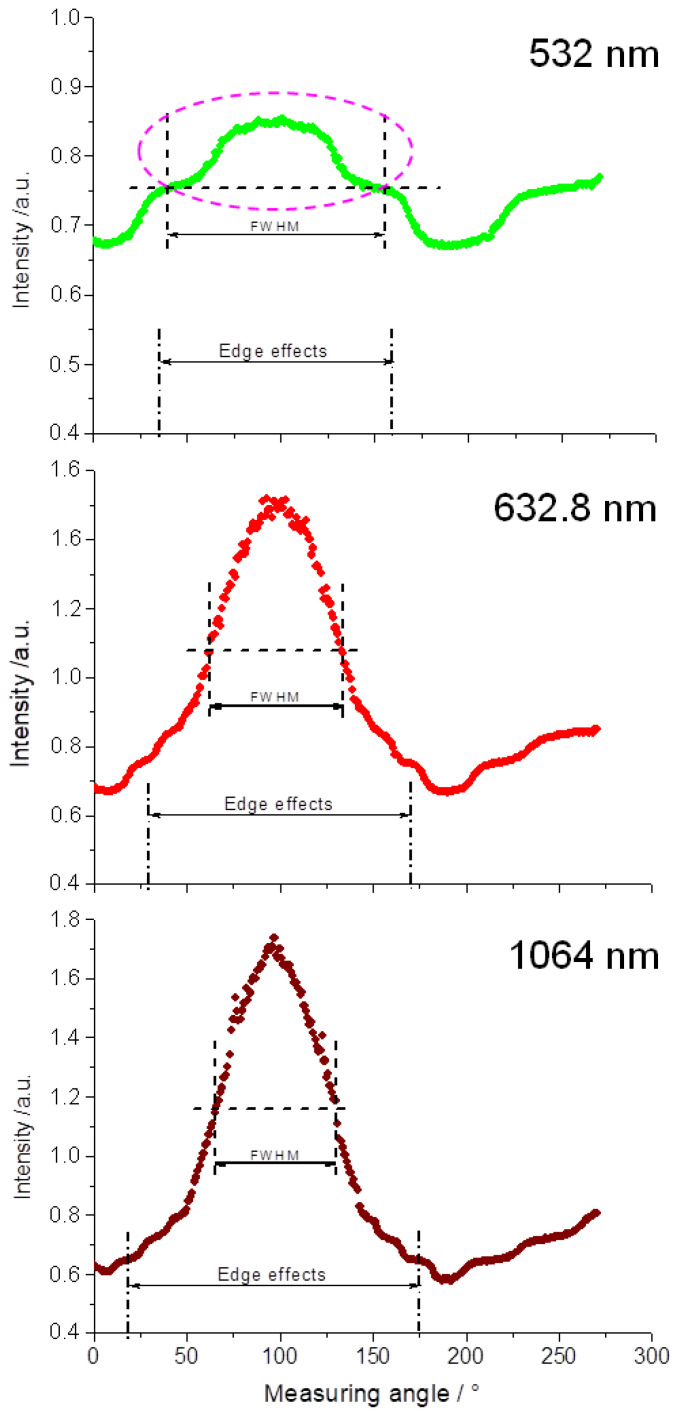
Line widths (FWHM) and edge effects for the various wavelengths measured on the ceramic base model. With increasing wavelengths, the line widths decrease whereas the edge effects increase. For the wavelength 532 nm, the line width and the width of the edge effects almost coincide. Therefore, the line width was determined above the edges (pink dashed oval).

Figure 5 shows the line widths for the various wavelengths concerning the base model. The edge effects, described in Figure 2, emerge most prominently at wavelength 532 nm in the area of the line width (FWHM). Even though these edge effects can be likewise seen at wavelengths 632.8 nm and 1064 nm, they do not affect the determination of the line widths. The different manifestation of these edge effects illustrates the angular widening Δ for the various wavelengths. Thus, at wavelength 532 nm, this edge effect occurs in the angular range of 35° and 160° (Δ = 125°). At wavelength 632.8 nm, an angular range of 30° and 170° (Δ = 140°) was determined, whereas an angular range of 20° and 175° (Δ = 155°) was ascertained for wavelength 1064 nm. Realizing this wavelength dependency of Δ_532 nm_ < Δ_632_._8 nm_ < Δ_1064 nm_ or conversely for the line widths (FWHM) 116°_FWHM @ 532 nm_ > 71°_FWHM @ 632_._8 nm_ > 65°_FWHM @ 1064 nm_ is decisive for the later interpretation of the results.

Since at wavelength 532 nm the line width overlaps with edge effects, the area above the edges were used for measuring the line width (FWHM) (Figure 5, pink dashed oval). All line widths could be calculated with a polynomial fit of the program OriginPro 8.6.

**Figure 6:**
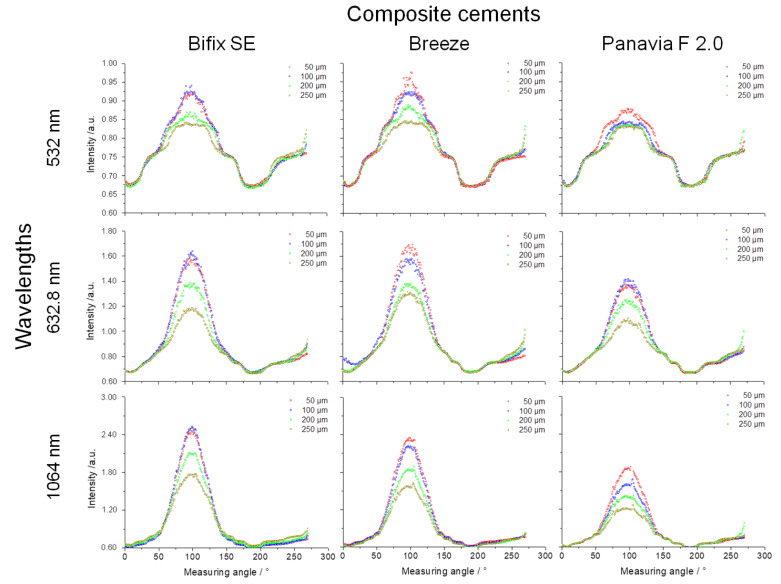
Intensity and line width (FWHM) diagrams, in the x-axis according to Figure 1 from “light” to “dark” cements, and in the y-axis, the wavelength dependency from short-wave (upper) to long-wave radiation (lower). The layer thicknesses are color-coded in the graphs.

According to Figure 1, the luting polymers (cements) were sorted from “light” to “dark” (Bifix SE → Breeze^TM^ → Panavia^TM^ F 2.0). The intensity and the line width diagrams (Full Width at Half Maximum) were likewise analyzed in this order (Figure 6). For every sample specimen, the line form, averaged over four measurements and depending on the wavelength and the cement layer thickness, was used for determining the respective line width (FWHM). The standard deviation values calculated here were below the 1‰ limit and, thus, were no longer sensibly presentable.

The comparison of the intensity of the measured lines confirms the transmission behavior from the power measurements (Figure 4) as follows:

- The light transmission decreases with increasing layer thickness.
- The light transmission increases with increasing wavelength.
- The light transmission decreases respectively depending on the material and layer thickness from “light” to “dark”.

The first two statements can be clearly verified by the measurement values from Table 2 for all examined cements. The last statement, however, is not fully congruent with the measurement values from Table 2. In the visual spectral range (532 nm and 632.8 nm), Breeze^TM^, compared to Bifix SE, depicts comparable or lightly increased transmissions. In the near-infrared range (1064 nm), the last statement can again be clearly substantiated. Being the visually “darkest” material, Panavia^TM^ F 2.0 shows the lowest intensities at all cement layer thicknesses and at all wavelengths.

**Figure 7:**
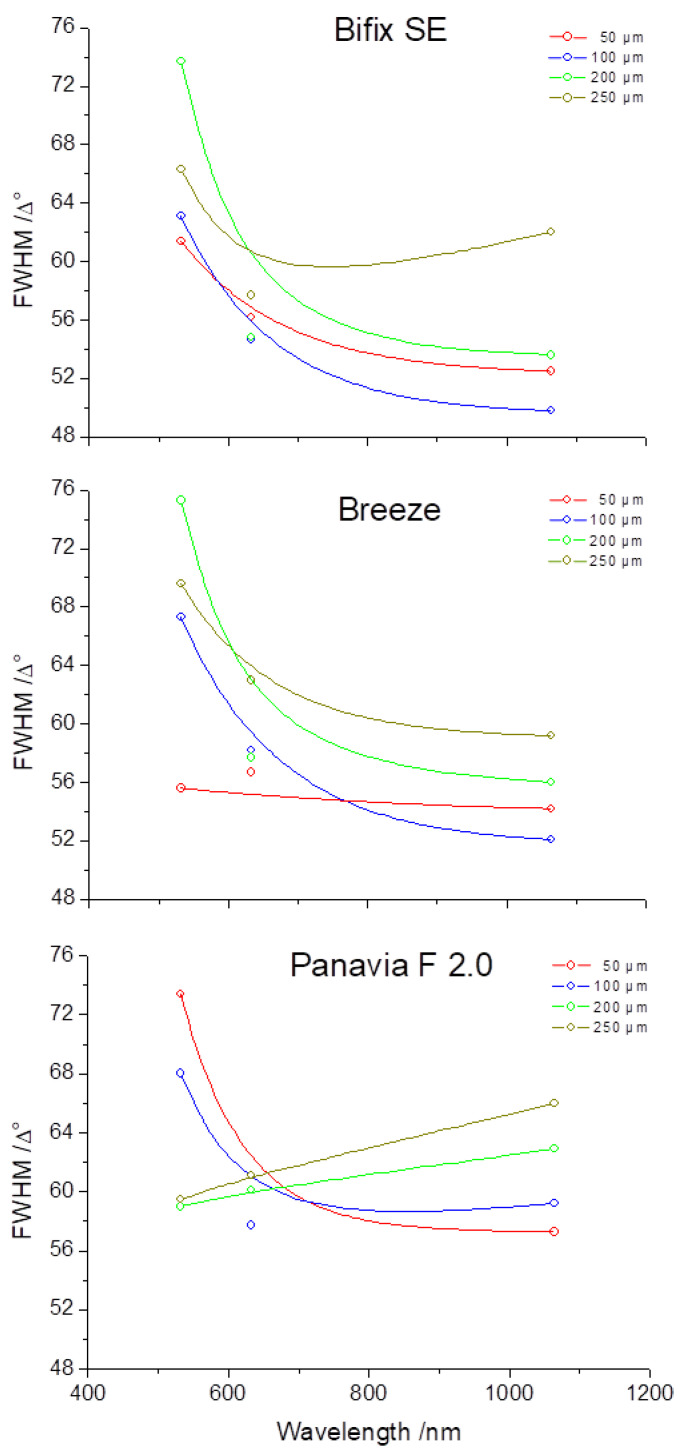
Schematic diagrams of the line widths (Full Width at Half Maximum (FWHM) of various cements as a function of the wavelength).

For the self-adhesive, dual-cure luting polymers Bifix SE and Breeze^TM^, the line widths (FWHM) show comparable curves at all layer thicknesses, whereas Panavia^TM^ F 2.0, likewise a dual-cure luting polymer but in a two-component mixing, displays considerable deviations in the course of the curves at layer thicknesses of 200 and 250 µm (Figure 7).

The following facts can be expressed for the individual layer thicknesses:

- At a layer thickness of 50 µm, it is observed that all luting materials show a clear trend towards a reduction of the line widths from the visual to the near-infrared spectral ranges.
- The reduction in the line widths at a layer thickness of 100 µm is likewise observed for all luting materials with the exception of Panavia^TM^ F 2.0 which shows a slight increase in the trend curve at wavelength 1064 nm.
- For Panavia^TM^ F 2.0 at a layer thickness of 200 µm, the increase in the line width is now discernible over the entire wavelength range.
- This trend for Panavia^TM^ F 2.0 is likewise seen at a layer thickness of 250 µm. However, also for Bifix SE at this layer thickness, there is an increase in the line width towards the near-infrared spectral range (1064 nm).

Moreover, the behavior of the line widths (FWHM) should be considered for the individual wavelengths:

- Noteworthy is that at wavelength 532 nm, Bifix SE and Breeze^TM^ show an increase in the line width at increasing layer thicknesses of 50, 100, and 200 µm. However, at a layer thickness of 250 µm, the line width again decreases. Yet, for Panavia^TM^ F 2.0, the line widths at layer thicknesses of 200 and 250 µm lie far below those of layer thicknesses 50 and 100 µm.
- For the wavelength 632.8 nm, all luting materials display comparable measurement values.
- Only for Panavia^TM^ F 2.0 can an increase in the line width be discerned with increasing layer thickness. For Bifix SE and Breeze^TM^, however, this behavior is reversed at layer thicknesses 50 µm and 100 µm.

## 4. Discussion

The goniophotometry represents a proven method for measuring angle-dependent scattered light distributions of a sample specimen. In this context, Jacques et al. [27] mentioned the problems of multiple scattering phenomena at corresponding layer thicknesses. At a layer thickness of the ceramic base model of 0.4 mm, edge effects caused by multiple scattering are clearly recognizable here (Figure 5). Since scattering represents a wavelength-dependent phenomenon ($I\;\sim \;\frac{1}{{}^{\lambda^{4}}}$), the edge effects are especially pronounced at 532 nm [28,29]. This was considered during the line width (FWHM) analyses.

According to realistic conditions, all the sample specimens were irradiated from the ceramic side. The losses resulting from the back reflection as well as from the pretreatment with 5% hydrofluoric acid before the cements applied were of a systematic nature and, thus, identical for all sample specimens. Therefore, the influence of surface reflection and scattering based on surface etching could be neglected for all sample specimens.

The laser output powers of the vanadate lasers (532 nm and 1064 nm) were calibrated to the maximum output power of the He-Ne-laser (632.8 nm). The calibration of the vanadate lasers, however, proved itself difficult in its lower power spectrum because of instabilities in the threshold range which resulted in a certain fluctuation margin of the emitted power (Figure 3). The power loss caused by the base models in the visible range was in the order of approximately 52%. In consideration of their wavelengths used, the losses through feldspathic ceramic are comparable with the results of Rasetto et al. [30].

### 4.1. Power and Intensity Measurements

**Figure 8:**
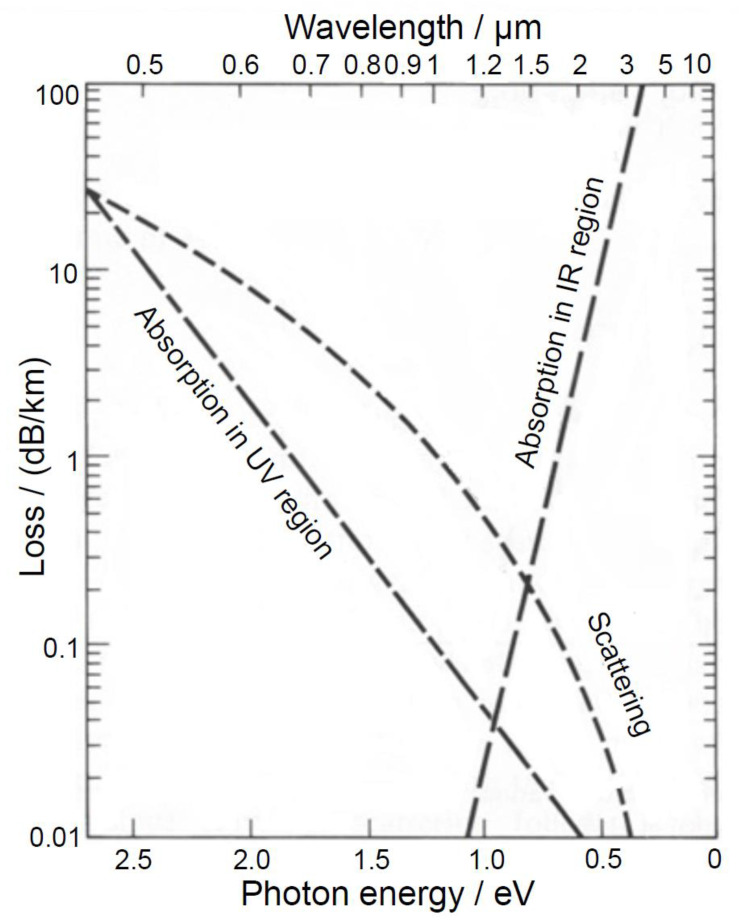
Diagram of the wavelength-dependent loss mechanisms of absorption and scattering (adapted from Horiguchi and Osanai [31] with permission from the Institution of Engineering and Technology (IET) 2023), presented here in units of dB/km. Regarding the studies here, the loss mechanisms are reflected in the changes in power, intensity, and line widths (FWHM).

The increase in power and intensity of the transmitted radiation with increasing wavelength (632.8 nm → 1064 nm) was observed for all luting materials and layer thicknesses (Figure 4 and Figure 6). This increase can be explained by the reduced influence of scattering effects towards the near-infrared range (Figure 8).

**Figure 9:**
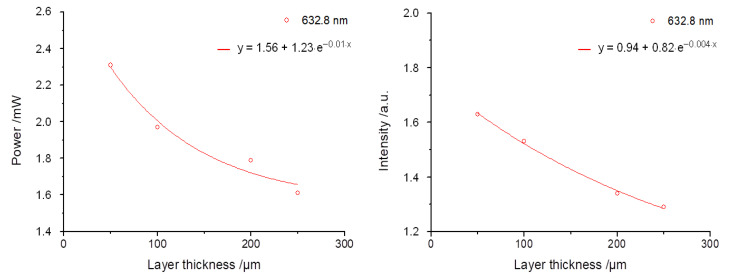
Exponential decrease in power/intensity exemplified for the sample specimen with the luting polymer Breeze^TM^ at wavelength 632.8 nm, presented as a function of the layer thickness.

In general, the sum of all loss mechanisms is designated as extinction ε. In turbid media, absorption and scattering are primary interactions, whereby ε is expressed by the following equation:

ε = µ_A_ + µ_S_, (1)

where µ_A_ is the absorption constant and µ_S_ is the scattering constant. The accompanying intensity losses can be interpreted according to the Lambert–Beer law as:

I = I_0_e^−εx^, (2)

where the term I denotes the transmitted radiation intensity and I_0_ the incident radiation intensity, ε the material-intrinsic absorptivity of the attenuating species, and x is the optical penetration depth. Figure 9 exemplarily shows the exponential decrease in power or intensity for Breeze^TM^ at 632.8 nm from the values of Table 1 and Table 2.

The various values for ε can be attributed to the differences in the measurement data acquisition. Due to the increased measurement angle for the power measurement at the detector (Power measurements and evaluation), not only the absorption, but also considerable scattering fractions, are detected, which leads to an ε of 0.01 µm^−1^ according to Equation (1). For the intensity measurement, the extinction results merely in ε = µ_A_ (0.004 µm^−1^), because the data are acquired over a very small spatial angle in the center (measuring angle at 100°, Figure 2) and, thus, the scattering fractions can be neglected. Consequently, this can explain the various ε-values on hand of the measurement procedure.

The fundamental material-specific differences can be illustrated from the intensity or line width diagrams (Figure 6). The luting polymers are composed of their organic polymer matrix, mixed with inorganic fillers and adjuvants such as initiators, catalysts, and inhibitors. The optical properties are enhanced by color pigments and the inorganic fillers. Therefore, the effect on the light interaction is based on the composition of the polymers. For example, the light transmission is affected by the differences in the refractive indices of the polymer matrix and the fillers [32]. Moreover, the monomer type as well as the number, kind, and form of the fillers affect the transmission [33,34,35]. The absorption occurs in the color pigments and/or polymer matrix, whereby the scattering is related to the fillers [36].

Bifix SE as well as BreezeTM are self-adhesive luting polymers and show an approximately identical perceived brightness (Figure 1). Thus, the transmission (Figure 6) and line width curves (Figure 7) are comparable for all laser wavelengths and layer thicknesses. On the other hand, since PanaviaTM F 2.0 application requires a separate adhesive to the tooth enamel, compared to both other luting polymers, PanaviaTM F 2.0 looks darker and somewhat yellowy (Figure 1). The transmissions seem to be clearly lower (Figure 6) and the line width curves definitely differ from one another at layer thicknesses 200 µm and 250 µm. Guiraldo et al. [37] reported about the reduction of light transmission at dark colors of conventional polymers, which is associated with increased absorption. The yellowing can be attributed to camphor quinone in the initiator system [38]. Furthermore, the mixing-in of the adhesive may adversely affect the mechanical properties [39]. However, the extent to which the optical properties are affected here is unknown.

**Figure 10:**
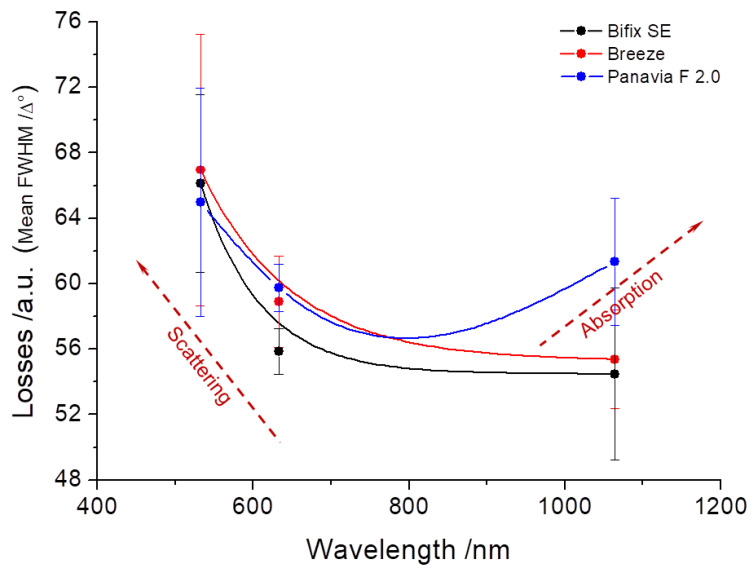
Mean line widths (FWHM) over all layer thicknesses for every wavelength taken from Figure 7. The trends of the loss mechanisms of scattering and absorption can be clearly categorized from Figure 8.

The courses of the curves for the various luting materials from Figure 7 can be interpreted by means of Figure 8. Figure 8 principally demonstrates the absorption and scattering behavior in the wavelength range of between 0.5 µm and 10 µm. Hereby, the losses in a logarithmic scale are reflected in the unit dB/km on the y-axis. The interpretations of the courses of the curves from Figure 7 likewise describe the effect of absorption and scattering over the line widths (position and course of the curve as a function of the wavelength) of the transmitted light within the luting materials. Thus, for the applied layer thicknesses used in this work in the µm-range, Figure 8 can be used to make a fundamental assertion about the effects of the loss mechanisms within the luting materials (Figure 10).

In turbid materials, scattering and absorption normally occur simultaneously but with different weighting, depending on the layer thickness and color. Because of its darker color, Panavia^TM^ F 2.0 shows increased absorption effects already as of a layer thickness of 100 µm (rise in the curves, Figure 7) [37]. By contrast, Bifix SE shows this effect first at a layer thickness of 200 µm. This tendency could not be observed for Breeze^TM^.

### 4.2. Clinical Considerations

The entire visual perception of a full-ceramic restoration is aesthetically influenced, starting from the base (subjacent tooth layer) [15,40] over the luting polymer (cement) and extending to the actual restoration material, the ceramic. The pretreatment of the ceramic and the tooth surface, particle abraded and/or acid etched [19,41], can, based on the created surface structure in combination with the luting polymer, influence the optical as well as the mechanical properties [19] of the full-ceramic restoration. All these aspects are of clinical relevance because teeth as well as the restorative materials are translucent [42].

Especially in the luting polymers, increased scattering effects on the incorporated fillers appear, whereby their size and concentration affect the light scattering [43,44,45]. Moreover, the absorption within the luting polymer increases with increasing layer thickness [43]. Some studies report about changes in the color of full-ceramic restorations depending on the color of the applied luting material [46,47,48,49], whereby the thickness of the applied ceramic exerts a decisive influence. Thus, Vichi et al. [40] observed color differences after cementation at ceramic thicknesses of 1 mm.

Therefore, in the application of veneers and crowns in the front-tooth area, a luting polymer may not distort the entire aesthetic impression. Thus, May et al. [50] and Prakki et al. [51] suggest an optimal layer thickness of the luting polymer of between 100 µm and 300 µm, whereas manufacturers specify thicknesses ranging from 10 µm to 30 µm. The present investigations show that at layer thicknesses of 50 µm and 100 µm of all applied luting materials, there is no effect on the aesthetics, i.e., on the color perception, e.g., by absorption in the visible spectral range (Figure 7), which the losses reflect over the averaged line widths in the visible spectral range (Figure 10). Therefore, the defined null hypothesis had to be rejected for layer thicknesses above 100 µm.

The accuracy in which a restorative procedure can be successfully applied depends on different material (tooth, luting polymer, and ceramic) properties as: translucency [52], color [34,36], thickness [30,53], and shade [49,53], respectively. Adapting the material combinations to the clinical situation shows finally the aesthetical appearance of a full-ceramic restoration.

## 5. Conclusions

This study illustrates the complex interrelationships in using ceramic materials as full-fledged restoration materials. The tooth base (subjacent tooth layer), luting polymer (cement), and ceramic altogether form a multilayered optical system that affects the whole aesthetic image of the restoration. Ultimately, it could be shown here that the cements applied in thin layers (50 µm and 100 µm) only show a redistribution of light by scattering, which does not cause any impairment in the color perception. Therefore, knowledge about the optical properties of the luting cements and their interaction with light are greatly important for ensuring an aesthetically satisfactory outcome for the restoration.

## Figures and Tables

**Table 1:** Transmitted power values as a function of material, layer thickness, and wavelength. Δ mW specifies the difference in values of the transmitted powers among the corresponding wavelengths, thus giving information about their increase.

	Layer Thickness	Power [mW]	Power [mW]	Power [mW]	Δ mW	Δ mW
		532 nm	632.8 nm	1064 nm	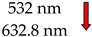	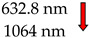
Bifix SE	50 µm	2.11	2.26	3.18	0.15	0.92
	100 µm	1.89	1.96	3.09	0.07	1.13
	200 µm	1.64	1.72	2.79	0.08	1.07
	250 µm	1.49	1.61	2.62	0.12	1.01
	Mean Δ mW				0.11	1.03
Breeze^TM^	50 µm	2.18	2.31	3.13	0.13	0.82
	100 µm	1.91	1.97	2.96	0.06	0.99
	200 µm	1.67	1.79	2.69	0.12	0.90
	250 µm	1.56	1.61	2.41	0.05	0.80
	Mean Δ mW				0.09	0.88
Panavia^TM^ F 2.0	50 µm	2.09	2.22	3.07	0.13	0.85
	100 µm	1.86	1.92	2.92	0.06	1.00
	200 µm	1.39	1.52	2.61	0.13	1.09
	250 µm	1.18	1.26	2.36	0.08	1.10
	Mean Δ mW				0.10	1.01

**Table 2:** Measured intensities and line widths (FWHM) as a function of material, wavelength, and layer thickness. The reported intensity values are the maximum values at the measuring angle 100° of the individual curves from Figure 6. The corresponding line widths were determined according to Figure 5.

		532 nm		632.8 nm		1064 nm	
	Layer Thickness	Intensity [a.u.] *	FWHM [Δ°]	Intensity [a.u.]	FWHM [Δ°]	Intensity [a.u.]	FWHM [Δ°]
Bifix SE	50 µm	0.92	61.4	1.56	56.2	2.46	52.5
	100 µm	0.93	63.1	1.57	54.7	2.52	49.8
	200 µm	0.86	73.7	1.32	54.8	2.10	53.6
	250 µm	0.84	66.3	1.14	57.7	1.77	62.0
Breeze^TM^	50 µm	0.95	55.6	1.63	56.7	2.35	54.2
	100 µm	0.92	67.3	1.53	58.2	2.22	52.1
	200 µm	0.88	75.3	1.34	57.7	1.88	56.0
	250 µm	0.84	69.6	1.29	63.0	1.62	59.2
Panavia^TM^ F 2.0	50 µm	0.88	73.4	1.33	60.1	2.04	57.3
	100 µm	0.84	68.0	1.37	57.7	1.67	59.2
	200 µm	0.83	59.0	1.20	60.1	1.41	62.9
	250 µm	0.83	59.5	1.06	61.1	1.27	66.0

*: a.u. for arbitrary units.

## Data Availability

The data presented in this study are available on request from the corresponding author.
